# Vascular disease persistence in giant cell arteritis: are stromal cells neglected?

**DOI:** 10.1136/ard-2023-225270

**Published:** 2024-04-29

**Authors:** Maira Karabayas, Hafeez E Ibrahim, Anke J Roelofs, Gary Reynolds, Dana Kidder, Cosimo De Bari

**Affiliations:** 1 Centre for Arthritis and Musculoskeletal Health, University of Aberdeen, Aberdeen, UK; 2 Centre for Immunology, Massachusetts General Hospital, Boston, Massachusetts, USA

**Keywords:** Giant Cell Arteritis, Vasculitis, Fibroblasts, Inflammation

## Abstract

Giant cell arteritis (GCA), the most common systemic vasculitis, is characterised by aberrant interactions between infiltrating and resident cells of the vessel wall. Ageing and breach of tolerance are prerequisites for GCA development, resulting in dendritic and T-cell dysfunction. Inflammatory cytokines polarise T-cells, activate resident macrophages and synergistically enhance vascular inflammation, providing a loop of autoreactivity. These events originate in the adventitia, commonly regarded as the biological epicentre of the vessel wall, with additional recruitment of cells that infiltrate and migrate towards the intima. Thus, GCA-vessels exhibit infiltrates across the vascular layers, with various cytokines and growth factors amplifying the pathogenic process. These events activate ineffective repair mechanisms, where dysfunctional vascular smooth muscle cells and fibroblasts phenotypically shift along their lineage and colonise the intima. While high-dose glucocorticoids broadly suppress these inflammatory events, they cause well known deleterious effects. Despite the emerging targeted therapeutics, disease relapse remains common, affecting >50% of patients. This may reflect a discrepancy between systemic and local mediators of inflammation. Indeed, temporal arteries and aortas of GCA-patients can show immune-mediated abnormalities, despite the treatment induced clinical remission. The mechanisms of persistence of vascular disease in GCA remain elusive. Studies in other chronic inflammatory diseases point to the fibroblasts (and their lineage cells including myofibroblasts) as possible orchestrators or even effectors of disease chronicity through interactions with immune cells. Here, we critically review the contribution of immune and stromal cells to GCA pathogenesis and analyse the molecular mechanisms by which these would underpin the persistence of vascular disease.

## Introduction

Giant cell arteritis (GCA), an age-related inflammatory disease of large and medium-sized arteries, predilects the temporal artery (TA) as well as the aorta and its major branches.^1^ It is the most common systemic vasculitis in adults with an incidence rate of 7–22 per 100 000 across Europe.^2^ GCA typically affects individuals over the age of 50 years and disease prevalence increases with advancing age, exhibiting marked female preponderance.^2 3^ Characteristic of GCA is the synergistic over-activation of the innate and adaptive immune systems, which manifests in systemic and vascular wall inflammation.^4^ These complex interactions result in luminal stenosis or aneurysmal wall damage dependent on tissue tropism, clinically translating in complications such as visual loss, stroke or aneurysm formation.^5–7^ Extensive research in GCA has primarily focused on immune cells and their effector pathways. Despite, licensed therapeutic options remain limited. High-dose glucocorticoid courses anchor standard care, with IL-6R blockade used as a steroid-sparing agent yielding mixed outcomes.^8–10^ The paucity of treatment choices reflects our limited understanding of the complex immunobiology of GCA, the role of immune and stromal interactions, and the mediators of vascular disease persistence beyond the acute phase.

Several studies have demonstrated aortic inflammatory infiltrates in GCA-treated patients undergoing surgery.^11–13^ Most cases exhibit mild-to-moderate inflammation and are likely affected by cohort bias.^11–13^ Nonetheless, these studies provide insights into persistent vascular inflammation, yet the precise nature of these lesions and their clinical significance remain largely unknown. Targeting vascular disease persistence poses an unmet clinical need in GCA, as this often links with refractory disease courses. Studies on other chronic autoimmune/inflammatory diseases indicate that tissue-resident cells play crucial roles in perpetuating inflammation, in synergy with immune cells.^14 15^ Here, we aim to (a) provide relevant introductory pathogenic mechanisms in GCA, (b) critically review the literature on the role of immune and stromal cells in sustaining vascular inflammation, (c) examine their interplay and (d) draw links to stromal biology derived from other chronic inflammatory diseases.

## Histopathology

Anatomically, large and medium-sized arteries are defined by (outside-in) the tunica adventitia, media and intima, separated by dense elastic laminae.^16^ The intima consists of endothelium and a thin subendothelial connective tissue, while the media is mainly composed of vascular smooth muscle cells (VSMCs). The outermost adventitial layer houses the vasa vasorum, vascular dendritic cells (vasDCs), fibroblasts and nerve endings.^16^

In GCA, primed vasDCs and T-cells, strategically positioned within the adventitia, act as early orchestrators of the inflammatory cascade.^17^ Interactions between infiltrating and resident cells ultimately deliver a maladaptive mural repair process.^17^ Histologically, this translates to an expanded adventitia, medial atrophy and VSMC loss, disrupted and often duplicated internal elastic lamina (IEL) and a hyperplastic neo-intima with newly formed microvascular networks.^18–21^ Inflammation can be either limited to the adventitia, extending into the media, transmural, or affecting the adventitia and intima with sparing of the media, also referred as the concentric ring or bilayer.^18 21^ It is increasingly appreciated that in GCA-vessels, resident stromal cells including VSMCs, fibroblasts and myofibroblasts, contribute to GCA pathogenesis and are not merely innocent bystanders.^22–24^ VSMCs express contractile proteins such as smooth muscle actin (*ACTA2*), heavy chain myosin (*MYH11*) and calponin (*CNN1*), provide arterial contractility and maintain vascular tone.^25^ In GCA, VSMCs proliferate, adopting a proinflammatory phenotype.^22 24^ Fibroblasts regulate ECM homeostasis^26^ and, when activated, can undergo phenotypic differentiation into myofibroblasts expressing ACTA2 but devoid of late differentiation VSMC markers such as MYH11 and CNN1.^27 28^ However, fibroblasts are not the sole precursors of myofibroblasts as VSMCs can also differentiate into myofibroblasts in vascular diseases.^29 30^ In this article, we will review the roles of these resident stromal cells in GCA pathogenesis.

## Ageing predisposes to GCA development

The elderly exclusivity of GCA indicates an aetiological link between ageing and disease development. Monti *et al* found age to be associated with a higher incidence of ischaemic complications and aortic aneurysm formation/dissection.^31^ Yet, more refractory disease has been reported in younger cohorts.^32^ Despite the association, the mechanisms of ageing in GCA remain to be elucidated.

**Figure 1:**
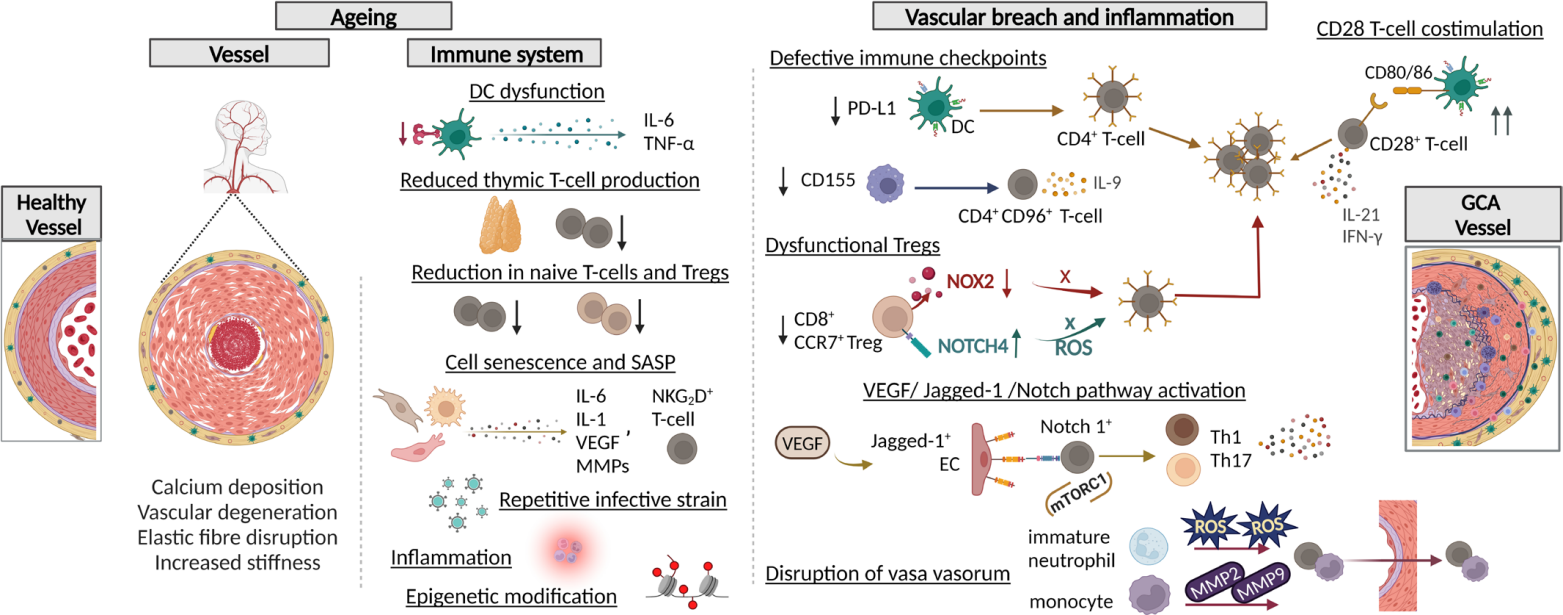
Ageing of the immune system and vessel wall and vascular breach leading to early inflammation. Vascular ageing and ageing of the immune system are key predisposing factors in GCA pathogenesis. The ageing vessel exhibits elastic fibre disruption and increased vascular stiffness. Further, immunosenescence is characterised by a reduction in thymic T-cell production, DC dysfunction and reduction in circulating naïve T-cells and Tregs. In addition, cellular senescence observed in fibroblasts endothelial cells and macrophages in GCA lesions, links with the production of proinflammatory cytokines as well as catabolic factors (MMPs and VEGF). Circulatory and adventitial CD4 and CD8 T-cells also demonstrate cell senescence. Repetitive infective strain on the immune system and inflammation associate with epigenetic modifications which are also linked to this process. Meanwhile, distinct mechanisms are implicated for vascular breach leading to early inflammation as observed in GCA vessels. These include. (i) Defective PD-1/PD-L1 immune checkpoints: GCA vasDCs express low levels of PD-L1 which results in recruitment, activation and retention of PD-1^+^CD4^+^ T-cells in GCA arteries. (ii) Defective CD155/CD96 immune checkpoint: GCA macrophages express low levels of the immunoinhibitory ligand CD155 and this allows for uncontrolled activation and proliferation of CD4^+^CD96^+^T-cells which infiltrate the vessel wall and produce effector cytokines (IL-9). (iii) Increased CD28 T-cell costimulation in GCA: there is aberrantly increased interaction between CD80/86 on vasDCs and CD28 on CD4^+^ T-cells leading to enhanced T cell effector functions and cytokine production (IL-21 and IFN-γ). (iv) Dysfunctional regulatory T-cells: aberrantly increased Notch4 signalling in GCA CD8^+^ Tregs leads to restriction of NOX2-exosomal release, hence failing to generate ROS required for modulation of CD4^+^ T-cell activity. (v) Aberrant activation of the VEGF/Jagged-1/Notch pathway. VEGF induces Jagged-1^+^ expression by ECs which then interact with NOTCH1^+^ T-cells resulting in T-cell polarisation (Th1/Th17). These polarised T-cells infiltrate and intensify vasculitic immune activity. (vi) Disruption of endothelial lining of the vasa vasorum due to increased production and activity of ROS and MMPs by immature neutrophils and monocytes, respectively. This culminates in immune cell infiltration. EC, endothelial cell; GCA, giant cell arteritis; IL, interleukin; MMPs, matrix metalloproteinases; NOX2, NADPH oxidase 2; ROS, reactive oxygen species; TNF-α, tumour necrosis factor alpha; Treg, regulatory T-cell; vasDC, vascular dendritic cell.

Vascular ageing manifests as medial atrophy, reduced VSMC plasticity, calcification of the IEL and elastic fibre disruption (figure 1),^33 34^ but whether these changes predispose to GCA is unclear. Ageing of the immune system is associated with a reduction in thymic T-cells, while accrued infective insults lead to a decline in naïve and regulatory T-cells (Tregs) and an expansion of memory and effector T-cells (figure 1).^33^ With advancing age, DC exhibit reduced toll-like receptor functions, increased production of proinflammatory cytokines and enhanced reactivity to self-antigens.^35 36^ Impaired tissue tolerance orchestrated partly by dysfunctional vasDCs is considered a pre-requisite for GCA development, while vasDCs have been shown to enhance their entrapment in GCA-lesions through chemokine production.^37^

Ageing is associated with an accumulation of senescent cells, linking with age-related diseases such as atherosclerosis.^38 39^ Senescent cells can adopt the so-called senescence-associated secretory phenotype (SASP), characterised by secretion of proinflammatory cytokines (interleukin (IL)-6, IL-1), growth factors (VEGF) and matrix metalloproteinases (MMPs), which damage the surrounding tissue and serve as further inflammatory priming (figure 1).^39 40^ Recent studies reported the presence of senescent fibroblasts, macrophages and endothelial cells (EC) in GCA-lesions, and identified cells representative of SASP.^41 42^ Previously, cell senescence was also demonstrated in circulating and infiltrating T-cells (CD4 and CD8) in the vasa vasorum of GCA-arteries.^43^

Ageing and inflammation favour epigenetic changes. GCA-lesions exhibit DNA methylation profiles associated with expression of proinflammatory genes correlating to T-cell polarisation (Th1/Th17), a characteristic GCA signature.^44–46^ Accordingly, Estupiñán-Moreno and colleagues identified variable methylation and gene expression profiles in circulating CD14^+^ monocytes from GCA patients.^47^ They further demonstrated that glucocorticoid therapy alters the epigenome and transcriptome, with downregulation of genes involved in pathogenic inflammatory signalling pathways as well as cell proliferation/migration and angiogenesis.^47^ They intriguingly showed no substantial differences when comparing treatment-free remission patients to controls.^47^ The observed epigenetic changes are therefore likely multifactorial, mediated by ageing, environment and inflammation, and influenced by therapies.

Finally, dysregulation of microRNAs has been implicated in vascular ageing and autoimmunity owing to their ability to regulate gene expression post-transcriptionally and ultimately modify cellular function. Several microRNAs are highly expressed in GCA-lesions,^48 49^ and some of them are associated with VSMC synthetic and contractile functions, proposing a role in vascular inflammation and remodelling.^49 50^ Ageing therefore predisposes towards autoimmunity and autoinflammation, thus posing as a susceptibility factor for GCA development.

## Vascular breach and inflammation

Several mechanisms have been implicated in the vessel wall breach in GCA (figure 1). These include dysfunctional tissue-protective immune checkpoints, such as the PD1-PDL1 and CD155-CD96 checkpoints, playing key pathogenic roles in breaching immune tolerance and enhancing T-cell recruitment and activity in GCA-arteries.^51 52^ In line with these, CD28-mediated T-cell costimulatory signals are active in GCA-arteries and promote T-cell proliferation, metabolic fitness and cytokine production, as demonstrated via CD28 inhibition in human artery-SCID chimaera mice.^53^ Additionally, CD28-mediated signalling promotes intimal hyperplasia and neoangiogenesis, but the mechanisms remain unclear.^53^ Furthermore, circulatory GCA Tregs fail to release reactive oxygen species (ROS) due to aberrantly increased Notch4 signalling.^54^ As such, their immunosuppressive function is deficient, resulting in unopposed CD4^+^ T-cell vascular infiltration and proinflammatory activity.^54^ Aberrant VEGF-induced Jagged1 expression by ECs of the vasa vasorum permits vascular infiltration of Notch1^+^CD4^+^ T-cells.^55 56^ The Jagged1-Notch1 interaction in CD4^+^ T-cells promotes Th1 and Th17 differentiation.^56^

Disruption of physical barriers of the vasa vasorum by monocytes and immature neutrophils also contributes to immune cell infiltration in GCA.^57 58^ Circulating monocytes and tissue macrophages express significantly higher amounts of MMPs MMP-2 and MMP-9 compared with age-matched controls,^57 59 60^ and MMP-9-producing monocytes appear to be a prerequisite for CD4^+^ T-cell invasion in studies using in-vitro assays and human artery-SCID chimaera mice.^57^ Furthermore, immature neutrophil populations (CD10^lo^CD64^–^CD16^hi^ and CD10^lo^CD64^+^CD16^lo^) present in GCA patients’ blood and TAs mediate vessel wall infiltration via increased production of extracellular ROS, increasing EC permeability as demonstrated via a neutrophil-endothelial coculture system.^58^

On entry into the adventitia, aberrantly activated CD4^+^ T-cells polarise into diverse T-cell subsets including effector T-cells with distinct cytokine expression profiles (interferon-gamma (IFN-γ), IL-9, IL-17, IL-21, IL-22 and GM-CSF producing T-cells).^44 46 52 61–63^ Cytokines produced by vasculitogenic T-cells promote further T-cell differentiation, activate macrophages and interact with stromal cells intensifying vascular inflammation. Tregs present in GCA-inflamed TAs exhibit plasticity, produce cytokines (IL-17) and contribute to sustaining vascular inflammation.^64^ FOXP3^+^ cells were found to coexpress IL-17A by double immunofluorescence staining of GCA-TAs.^64^ However, the specificity of FOXP3 expression to Tregs is unclear as other cell types can express FOXP3 when activated.^65 66^ For more details on vasculitogenic T-cells in GCA, we refer to the review article by Watanabe *et al*.^67^

**Figure 2:**
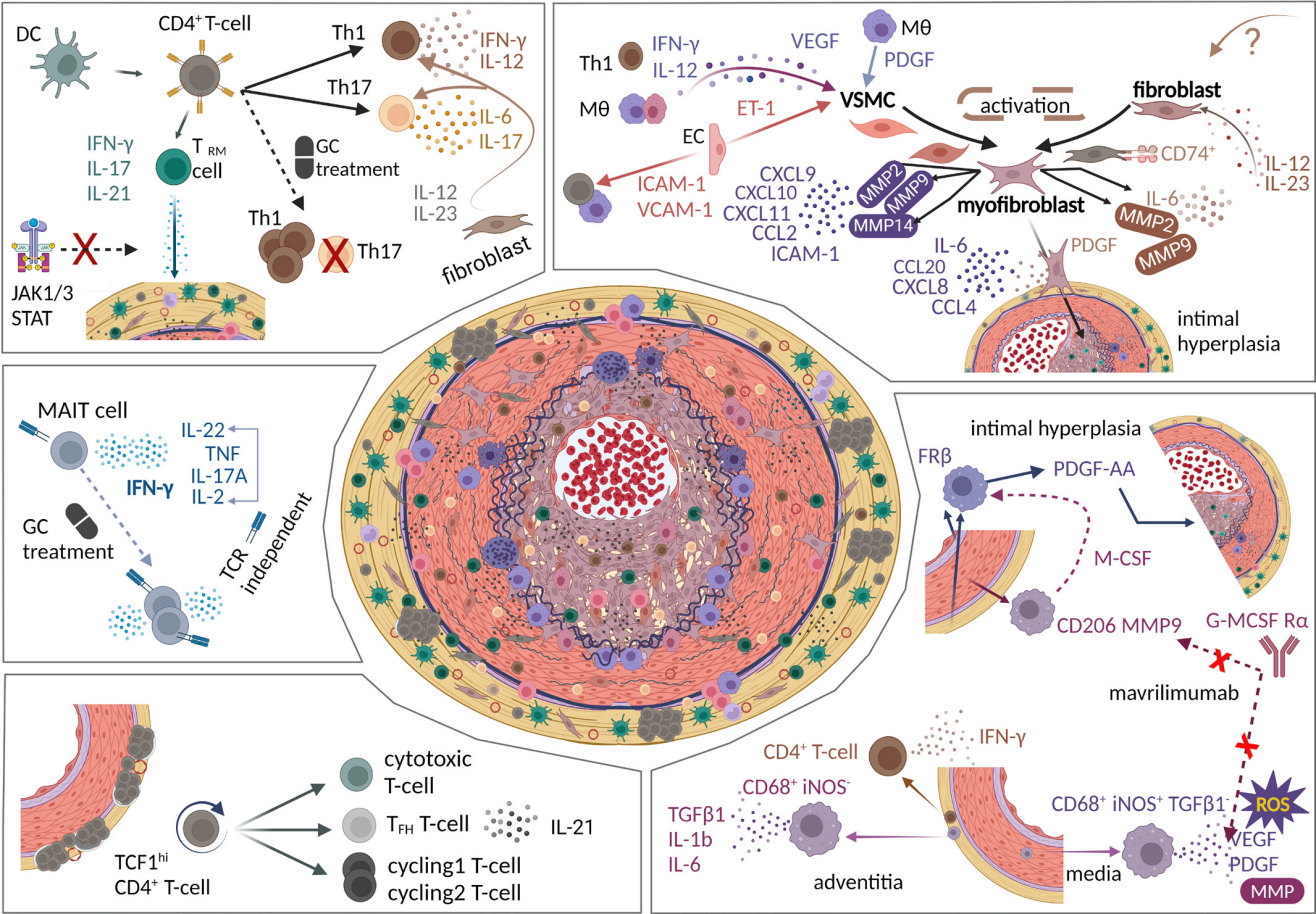
Vascular remodelling and maintenance of vascular disease activity. Clockwise: (i) T-cells are polarised into Th1 and Th17 cells with distinct cytokine profiles, with Th1 arm being steroid unresponsive. T_RM_ cells are also present in giant cell arteritis (GCA) vessels, but amenable to JAK-STAT inhibition. Fibroblast mediated by cytokine production promote T-cell polarisation. (ii) VSMCs, fibroblasts are activated by local cytokine milieu pathogenically transform along a differentiation axis to myofibroblasts and contribute to intimal hyperplasia. They are associated with the production of proinflammatory cytokines, chemokines and proteinases. GCA Fibroblasts express CD74^+^ suggestive of sentinel-like abilities and role in vascular disease activity. (iii) GCA tissue macrophages are heterogenous and spatially distributed in the vessel wall. The macrophage subtypes secrete distinct cytokine and growth factor profiles contributing differentially to the disruption of internal elastic lamina and intimal hyperplasia. (iv) The stem-like TCF1^+^CD4^+^ T-cells exhibit self-renewal capabilities and differentiate into cytotoxic T-cells, T_FH_ T-cells and cycling 1 and cycling 2 T-cells promoting vascular inflammation. (v) MAIT cells are present in GCA-affected vessels, proinflammatory (IFN-γ secretion) and are steroid unresponsive. DC, dendritic cell; GC, glucocorticoid; IFN-γ, interferon-gamma; IL, interleukin; MAIT cell, mucosal-associated invariant T cell; MMP, matrix metalloproteinase; MΘ, macrophages; ROS, reactive oxygen species; TCF1^+^CD4^+^ T-cells, T cell factor 1 expressing CD4^+^T cells; TCR, T cell receptor; T_RM_, tissue resident memory cells; VSMC, vascular smooth muscle cell. Figures were created using BioRender.com

In GCA-arteries, infiltrating monocytes differentiate into tissue macrophages and multinucleated giant cells (MNGCs). Tissue macrophages in GCA-TAs are associated with a CD68^+^CD16^+^CX3CR1^+^CCR2^−^ signature,^68^ and map within the arterial layers (figure 2).^69^ Medial and intimal macrophages produce MMPs, ROS, VEGF and PDGF and localise to the media/media-intima border proposing links with tissue destruction and vascular remodelling.^19 69–72^ Conversely, adventitial macrophages (CD68^+^iNOS^−^) produce TGF-β1, IL-1b and IL-6, and colocalise with IFN-γ-producing CD4^+^ T-cells, inferring a role in early GCA immunopathogenesis.^69^ Jiemy and colleagues identified two macrophage subtypes (FRβ^+^ and CD206^+^MMP-9^+^) in TAs via immunostaining.^73^ FRβ^+^ macrophages occupied predominantly the intima and adventitia, while CD206^+^MMP-9^+^ macrophages localised in the media and borders (figure 2).^73^ CD206^+^MMP-9^+^ macrophages, skewed by GM-CSF, appear tissue destructive and express M-CSF likely stimulating adjacent FRβ^+^ macrophages. The presence of FRβ^+^ macrophages correlates with the degree of intimal hyperplasia and PDGF-AA production.^73^

GM-CSF and GM-CSFRα are highly expressed by lesional macrophages, ECs and intimal myofibroblasts in GCA-TAs.^63^ Using TA ex-vivo culture models, Mavrilimumab, an anti-GM-CSFRα antibody, reduced proinflammatory cytokine production (IL-6, IL-1B and tumour necrosis factor alpha (TNF-α)), MMP-9 production and expression of CD206 and CD163 macrophage markers in GCA-arteries.^63^ Interestingly, in a phase 2 clinical trial, GCA patients exhibited a 27.4% lower incidence of relapse at 26 weeks and a sustained remission rate of 83%, 33.3% higher than the placebo group.^74^ This suggests that targeting dysregulated macrophages may be key in GCA management.

Neutrophils localise in the adventitia of GCA-vessels^58 75^ and associate with the formation of neutrophil extracellular traps decorated by IL-6 and IL-17A.^75^ Additionally, ECs lining neovessels in the adventitia and intima-media border also contribute to the propagation of inflammation. They express adhesion molecules (ICAM-1 and VCAM-1), which interact with corresponding receptors (Mac-1, LFA-1, VLA-4) on lesional lymphocytes and macrophages, suggesting EC-mediated transmigration of immune cells.^76^ The adhesion molecule-receptor interaction is promoted by S100 proteins, which are significantly expressed in GCA-vessels by infiltrating monocytes and neutrophils.^77^ Furthermore, IL-23p19-mediated gp130-STAT3 signalling in ECs upregulates the expression of adhesion molecules on ECs with subsequent promotion of lymphocyte transmigration.^78^ Taken together, these findings highlight the proinflammatory roles of ECs and neutrophils in propagating vascular inflammation in GCA.

## Vascular remodelling

GCA is characterised by uncontrolled vascular inflammation and tissue remodelling. In GCA-lesions, tissue destruction, effected by macrophages and their derivatives, is counterbalanced by the local production of growth factors promoting tissue repair via interaction with resident stromal cells (figure 2).^19 70 79^ However, this process becomes maladaptive leading to intimal hyperplasia and neoangiogenesis.^19 79^

GCA-vessels exhibit a proteolytic environment with increased MMP levels (MMP-2, MMP-9, MMP-14) exceeding their inhibitors (TIMP-1, TIMP-2), resulting in IEL degradation.^59^ MMPs are mainly produced by resident macrophages and MNGCs, but also by VSMCs and myofibroblasts.^71 80^ Using human artery-SCID chimaera mice, antibody-mediated MMP-9 inhibition significantly reduced intimal thickness and neovascular density, with reduction in VEGF, PDGF and FGF levels.^80^ Furthermore, macrophages produce ROS causing oxidative damage via lipid peroxidation in VSMCs in the media.^81^ The released ROS regulate the activity of MMPs by cleaving pro-MMPs to active forms, further enhancing tissue damage.^71 81^

Neovascularisation is typically observed in the media and intima of GCA-arteries.^19^ It is considered a compensatory mechanism to vascular hypoxia whereby new blood supply develops to support the expanded artery. VEGF, an angiogenic factor, is locally produced by macrophages and MNGCs at the media-intima junction, a site of neovascularisation.^19^ A positive correlation between the degree of neovascularisation and lesional VEGF expression has been observed in GCA.^19^ GCA-tissue macrophages also produce additional pro-angiogenic factors including angiogenin, PDGF and GM-CSF, which further contribute to neoangiogenesis.^22 63^

In GCA-arteries, VSMC dysfunction has been observed in response to mitogenic signals such as PDGF.^22^ GCA-TAs show transmural PDGF expression, predominantly at the media-intima junction, with levels correlating with intimal hyperplasia and clinical ischaemia.^72^ In-vitro studies demonstrated that PDGF induced the proliferation and migration of GCA-derived VSMCs.^22^ In line with this, imatinib (a tyrosine-kinase inhibitor inhibiting PDGF receptor activity) decreased myointimal cell proliferation, outgrowth, as well as production of collagen I/II and fibronectin in ex-vivo GCA models.^22^

Endothelin-1 (ET-1), a potent vasoconstrictor, is primarily secreted by ECs. However, in pathological states, ET-1 is also released by macrophages and VSMCs associating with tissue remodelling.^82 83^ GCA-lesions express high levels of ET-1 and its receptors ETAR and ETBR.^82^ In matrigel, ET-1 promotes VSMC motility through activation of focal adhesion kinase, while ET-1 receptor blockade reduces VSMC proliferation and migration.^84^ ET-1 exposure further stimulates MMP-2 production by VSMCs through ETBR signalling, indicating that ET-1 not only induces migratory effects on VSMCs but also enhances their proteolytic profile.^82^ Intriguingly, transmural expression of ET-1 along GCA-TAs correlates with a refractory clinical course associated with higher glucocorticoid treatment following 6-month follow-up.^84^ In a small cohort (n=3) of paired biopsies (46–50 weeks interval), despite a drop in ET-1 levels, ET-1 receptors remained partially elevated.^83^ Collectively, these data highlight that ET-1 might have targetable value in refractory disease.

In a longitudinal study, a small cohort underwent repeat TA biopsies following 1 year of treatment with infliximab alongside glucocorticoids.^85^ Interestingly, although mRNA expression of inflammatory cytokines (IL-1b, IL-6, IFN-γ, ICAM1) decreased, factors associated with vascular remodelling (PDGFA, PDGFB, TGF-β, MMP-9) increased. Similarly, in ex-vivo GCA-TA models dexamethasone reduced inflammatory gene expression but vascular remodelling associated factors (PDGRF, MMP-2, collagen I/III) were not glucocorticoid responsive.^86^ Collectively, these findings support the notion that VSMC plasticity remains pathogenic in late refractory disease.

## Vascular disease persistence

In GCA, persistence of vascular inflammation and disease activity despite treatment remains a clinically unmet need. On vessel wall entry, infiltrating CD4^+^ T-cells polarise into Th17 cells promoted by IL-6, IL-1β and IL-23 or Th1 cells mediated by IL-12 and IL-18. Th17 cells dominantly produce IL-6 and IL-17 and appear acutely steroid responsive, while Th1 reactivity, characterised by IL-12 and IFN-γ production, seems steroid resistant associating with disease chronicity.^44^ Intriguingly, in a cohort of GCA-treated patients undergoing repeat TA biopsies up to a year from diagnosis, lymphocytic infiltration persisted despite clinically quiescent disease.^87^ Similarly, Visvanathan *et al*, demonstrated high IL-12/23p40 and IFN-γ mRNA expression in 1-year post-treatment TAs of GCA patients with relapsing disease,^85^ while Deng *et al*, through repeat biopsies at 3–9 months, showed IFN-γ persistence despite treatment.^44^ These studies potentially suggest a role for Th-1 cytokines in disease chronicity, yet the exact mechanisms by which T-cell subsets exert their effector functions and their synergistic partners remain elusive. Furthermore, studies employing transcriptomic analyses of GCA and non-inflamed aortic tissues revealed an enrichment of interferon (I, II and III) signatures and JAK/STAT signalling in GCA.^88 89^ Pharmacogenomic network analysis also identified IFN-γ and CXCL10 to be druggable candidate genes.^89^ It is however noteworthy that disease duration prior to surgery is not specified for this cohort and the representative GCA-aortic cohort was relatively small.^89^

Despite the steroid-resistant Th1-related reactivity observed in GCA tissue, clinical studies targeting TNF-α and IL-12/IL-23p40 produced conflicting results.^90–93^ Meanwhile, ex-vivo work suggests that IFN-γ expression can be modulated by glucocorticoid treatment post-transcriptionally.^86^ Collectively, these findings highlight the complexity of T-cell signatures in GCA and the variability between studies. More in-depth and unbiased analyses of cellular heterogeneity in GCA-tissues from robust longitudinal studies are required to uncover the drivers underpinning vascular disease persistence. The pathogenic mechanisms involved in early inflammation likely contribute to this. However, there are other mechanisms that promote disease chronicity and persistence (figure 2).

### Tissue-resident memory T-cells

Tissue-resident memory T-cells (T_RM_ cells), a subset of CD4^+^ T-cells, are unable to recirculate from their resident tissues, can recruit various immune cells and elicit Th1, Th2 or Th17 effector functions on reactivation.^94 95^ Owing to their tissue residency and behaviour, T_RM_ cells are increasingly assessed for their roles in disease relapse in several autoimmune diseases. Zhang and colleagues identified a population of CD4^+^CD103^+^ T_RM_ cells within T-cells isolated from vasculitis-induced arteries developed via the human artery-SCID chimaera mice.^96^ These cells were only detected in trace amounts in peripheral blood. The group developed a trans-engraftment model whereby vasculitis-induced arteries from the human artery-SCID chimaera mice were trans-engrafted into SCID mice, but without transfer of PBMCs from GCA patients.^96^ This model was designed to explore the source and survival of T_RM_ cells in vasculitis-induced arteries by cutting off circulatory T-cell recruitment. They demonstrated a significant increase in T-cell density, infiltration and cytokine expression (IFN-γ^+^, IL-17^+^, IL-21^+^).^96^ The T_RM_ cells persisted following trans-engraftment signifying that these were not continuously recruited from the periphery, but survived and persisted in inflamed vessels.^96^ The lesional survival, maintenance and cytokine profile (IFN-γ^+^, IL-17^+^, IL-21^+^) of these cells were dependent on JAK1/3-STAT signalling.^96^ Recent work applying bulk RNA sequencing on GCA-inflamed aortic aneurysms revealed T_RM_ cell signatures to be enriched in GCA aortas versus controls.^97^ This not only validates their presence in GCA-arteries but further suggests they have a role in disease persistence, given the likely prolonged disease state of the obtained tissues. It is noteworthy that CD28 costimulation, which promotes T-cell glucose utilisation, supports the survival of T_RM_ cells.^53^ The presence, survival and profile of T_RM_ cells in GCA-arteries raises questions regarding their role in maintaining vascular inflammation and, by extension, GCA relapse.

### Stem-like T CD4^+^ T cells

GCA-inflamed aortas contain dense mononuclear aggregates organising in tertiary lymphoid structures (TLS) around adventitial microvessels.^97^ Recently, Sato and colleagues revealed adventitial TLS to contain a distinct T-cell subset, T-cell factor 1 expressing CD4^+^T cells (TCF1^+^CD4^+^ T-cells).^97^ Single-cell RNA sequencing of T-cells isolated from stripped adventitial layers of vasculitis-induced human TAs developed in SCID mice, delineated a CD4^+^ T-cell cluster expressing TCF7 (encoding the transcription factor TCF1) and lymphoid enhancer-binding factor 1 (LEF1, homologue of TCF1).^97^ This cluster was further enriched for T-cell survival associated genes (IL-7R), costimulatory (CD28, CD27, FAS) and TGF-β signalling (TGF-β, SMAD3) molecules.^97^ Collectively, these profiles support T-cell survival, self-renewal and stemness capabilities of TCF1^+^CD4^+^ T cells. Furthermore, TCF1^hi^CD4^+^ T-cells are likely central in TLS development as they express lymphoid development related genes (lymphotoxin beta, LTB) and lymphoid homing chemokine receptors (CCR7, CXCR3, CXCR5).^97^ Pseudotime analysis proposed the TCF1^hi^CD4^+^ T-cell cluster transitions into effector T-cells populations: cytotoxic CD4^+^ T-cells, IL-21-producing T follicular helper-like cells and cycling CD4^+^ T-cell clusters.^97^

IL-7R^+^TCF^hi^T cells were used to induce vasculitis in a human TA transplant SCID mouse, and the resulting vasculitis-induced TAs were serially trans-engrafted into a second SCID mouse without GCA PBMC transfer.^97^ The IL-7R^+^TCF1^hi^ T-cell-induced vasculitis exhibited significantly more proliferative T-cells, vascular inflammation, vascular injury and neovessel formation compared with the IL-7R^+^TCF1^lo^ T-cell induced vasculitic vessels.^97^ TCF1^hi^CD4^+^ T-cells sheltered in adventitial TLS likely contribute to vascular disease persistence by continued supply of pathogenic effector T-cells, which promote vascular inflammation and vascular remodelling in GCA-vessels.

### Mucosal-associated invariant T cells

Mucosal-associated invariant T (MAIT) cells, an innate-like T-cell subset, on activation proliferate and elicit rapid effector functions via production of proinflammatory cytokines.^98 99^ MAIT cells (CD3^+^IL-18R^+^TCRVα7.2^+^) are present at the media-adventitia border of GCA-TAs, but absent in controls.^100^ The percentage of circulating MAIT cells in GCA patients and controls is similar; however, GCA MAIT cells have higher proliferative capacity and are proinflammatory expressing high levels of IFN-γ.^100^ MAIT cells, activated by IL-12 and IL-18, produce IFN-γ via TCR-independent mechanisms, and these IFN-γ^+^ MAIT cells are not steroid responsive.^100^ The lack of correlation between CRP levels and proportion of circulating IFN-γ^+^ MAIT cells at baseline and after 3 months of glucocorticoid treatment suggests that these cells are disconnected from systemic inflammation, but relevant to vascular disease. While these single-centre study observations require further validation, they propose an important link between MAIT cells and lesional inflammatory persistence.

### Proinflammatory roles of VSMCs

Beyond vascular remodelling, VSMCs are appreciated for the inflammatory responses they elicit in pathological states. VSMCs express pattern-recognition receptors (TLR3 and TLR4) and secrete proinflammatory molecules in response to the cytokine milieu.^101 102^

GCA-derived VSMCs spontaneously exhibit upregulation of CXCL9 and CCL4 (mRNA) in-vitro, and secrete high levels of CXCL9 and CXCL10 as compared to control VSMCs, where expression appears virtually undetectable.^24^ Furthermore, in-vitro GCA TA-derived VSMCs produce high levels of CXCL9, CXCL10, CXCL11, CCL2 and intracellular ICAM-1 in response to IFN-γ or PDGF stimulation, thereby facilitating monocyte and T-cell chemotaxis and transmigration (particularly Th1).^22 24^ These chemokines (CXCL9, CXCL10, CXCL11), induced by IFN-γ, likely recruit CCR3^+^CD8^+^ T-cells into the arterial wall.^24 103^ Lesional CCR3^+^CD8^+^ T-cells in GCA-vessels elicit cytotoxic activities via granzyme B and TiA1 expression.^103^ Interestingly, PDGF inhibition via imatinib reduces VSMC CCL2 production in culture,^22^ indicating that VSMC-induced T-cell chemotaxis could be manipulated by PDGF inhibition, at least in-vitro. The proinflammatory reprogramming VSMCs undergo in response to effector functions of resident and infiltrating immune cells, demonstrates the bidirectional relationship between these cell types in GCA-arteries, resulting in a proinflammatory amplification loop. VSMCs thus contribute to the inflammatory events observed in GCA and are not merely targets of vascular remodelling.

Samson and colleagues generated ex-vivo ‘human monocyte-derived suppressive cells’ (HuMoSCs) and demonstrated that GCA-TA explants cultured with HuMoSCs or their supernatant exhibited a reduction in proinflammatory transcripts (CCL2, CXCR3, CCR2) as well as transcripts associated with ECM deposition (FN1, COL1A1, COL3A1), vascular remodelling and angiogenesis (PDGF, VEGF).^104^ Further, PDGF-AB treated VSMCs exhibited reduced migrative and proliferative capabilities in the presence of HuMoSCs supernatant, mediated by modulation of mTOR activity.^104^ These early observations introduce HuMoSCs for targeting dysfunctional GCA-VSMCs.

### VSMC, fibroblast and myofibroblast spectrum

Myofibroblasts exhibit diverse cellular origins.^105–107^ Lineage tracing and scRNA-seq studies have demonstrated in atherosclerosis and pulmonary artery hypertension that myofibroblasts derive from VSMCs, fibroblasts or both, suggesting that the underlying pathogenic mechanisms and tissue microenvironment determine which cells ultimately undergo phenotypic modulation.^29 30^

Parreau *et al* investigated the spatial distribution of fibroblasts across arterial layers of GCA and controls using immunohistochemical methods.^108^ A significant enrichment of myofibroblasts (CD90^+^α-SMA^+^) was observed in the adventitia and intima of GCA-TAs, correlating with the degree of hyperplasia.^108^ These were noted to form invading wells in areas of the damaged media.^108^ Myofibroblasts appeared to localise with inflammatory infiltrates^108^ and, given their proximity, interactions between these cell types are likely to occur. Strengthening this, Greigert *et al* identified a positive feedback loop by which myofibroblasts (CD90^+^, a-SMA^+^, MYH11^+^) located in the neointima of GCA-arteries, in response to IFN-γ and TNF-α, produce Th1/Th17 polarising cytokines (IL-12/IL-23) thereby sustaining T-cell polarisation.^23^ This suggests a mechanism by which stromal cells contribute to maintain the inflammatory process of vascular lesions, through interactions with immune cells. Further, cytokines such as IL-12 and IL-23, produced by resident and infiltrating immune cells in GCA-arteries, significantly increased myofibroblast outgrowth ex-vivo.^109^ These data imply that various cytokines are involved in promoting this invasive myofibroblast phenotype observed in GCA-lesions.^109^ However, the cell type of origin of myofibroblasts is unclear as they might derive from VSMCs, fibroblasts or both.

Phenotypic differentiation as well as migration of adventitial (myo)fibroblasts towards the intima and their contribution to neointima formation have been demonstrated using in-vivo animal models.^110–112^ Using murine vascular injury models, two groups demonstrated that labelled fibroblasts migrated from the adventitia to the intima, acquiring α-SMA expression during their migration.^111 112^ Findings of fibroblast functions observed in mechanical/endoluminal vascular injury and adventitial inflammation require validation in GCA. Nonetheless, a study revealed highly proliferative (Ki-67^+^) α-SMA^+^ cells to be enriched in the adventitia and intima of vasculitis-induced human arteries engrafted in SCID mice.^96^ Their proliferative activity significantly decreased on Tofacitinib (JAK1/3 inhibitor) treatment, with a notable reduction in intimal hyperplasia.^96^ Together, these data suggest that fibroblasts in GCA-arteries are likely activated in the adventitia, migrate towards the intima, and contribute to the intimal myofibroblast population and intimal hyperplasia. Since the adventitia is considered the biological epicentre of the vessel wall which tightly regulates vessel wall homeostasis and houses key events in the early GCA pathogenesis, the increased proportion of adventitial fibroblasts raises questions regarding their role in GCA inflammation beyond vascular remodelling.

Interestingly, thymic medullary fibroblasts in mice produce distinct self-antigens and express lymphotoxin-β receptor (LTβR), which is central for T-cell tolerance.^113^ LTβR deletion in these fibroblasts resulted in spontaneous loss of immune tolerance in murine models, with autoantibody production observed in multiple organs, associated with T-cell expansion.^113^ This suggests that fibroblast subsets have sentinel-like abilities which might also be reflective in GCA-vessels. In a recent study using spatial transcriptomics, CD74 (HLA class-II histocompatibility antigen) was identified as the most differentially expressed gene across all arterial compartments between GCA-arteries (n=9) and controls (n=7).^114^ Additionally, immunofluorescence staining showed CD74 to colocalise with the fibroblast marker CD90 in the media-intima border.^114^ Greigert *et al* also demonstrated that myofibroblasts significantly expressed HLA-DR, compared with controls.^23^ Stromal cells can express MHC class II molecules in response to inflammatory stimuli^115 116^; however, whether they have the capacity to induce T-cell proliferation analogous to conventional antigen-presenting cells remains unclear.

ScRNA-seq analyses of aneurysmal and non-aneurysmal aortas identified six fibroblast and seven VSMC clusters, with certain clusters exhibiting disease specificity.^117^ Aneurysmal aortas shared an expansion of the FB-S3 cluster, which appeared to hold an activated, profibrotic and proinflammatory phenotype, while FB-S1 was exclusive to control arteries.^117^ Transcriptomic analyses of (myo)fibroblast subsets in GCA-lesions and comparisons between TA and aortas could provide insights to endotypic classification.

Several studies have uncovered inflammation-associated fibroblast subtypes that promote or modulate inflammation in chronic inflammatory diseases.^14 15 118^ A study on rheumatoid arthritis integrating available scRNA-seq datasets of synovium, linked fibroblast phenotypes to disease pathotypes further correlating with disease severity indices.^119^ Perturbed fibroblast subsets were significantly expanded in inflammatory bowel disease tissues, and certain subsets correlated with clinical TNF-resistance.^118 120^ Emerging studies in GCA begin to uncover the immunomodulatory roles of the stroma. The investigation of cell heterogeneity in GCA-lesions could not only unravel disease mechanisms, but also inform disease stratification and targeted therapies.

## Challenges in studying GCA pathogenesis

Although existing functional models enhance our understanding of GCA pathogenesis, accurately recapitulating the GCA environment in animal models or functional assays remains challenging. Traditional mouse models are limited anatomically by the lack of vasa vasorum, the port of influx of circulating immune cells in GCA-lesions, while murine arteries unlikely benefit from the same immune tolerance that human medium and large arteries do. Further, SCID chimaera models fail to adequately depict the complexity of the human immune system including the effects of ageing. Pathogenic observations derived from GCA-lesions have been based on biased tissue methodologies such as immunostainings and focused qRT-PCR analyses, or targeted yet artificial in-vitro and ex-vivo culture models. This is particularly challenging for tissue-resident cells such as stromal cells, which by nature are elusive to investigate out-with their diseased environment. Yet, advancements in tissue transcriptomics expose cell heterogeneity in several immune and fibrotic disorders as well as cancers. These discoveries have translational value as they begin to shed light on disease stratification carving the path towards identification of novel targets. To systematically decipher the key cellular players responsible for disease chronicity in GCA, combination approaches and sophisticated technologies ought to be used with inherent cross-validation capabilities.

Moreover, as detailed in the comprehensive review by Tomelleri *et al*, an emerging school of thought suggests that cranial-GCA, PMR and LV-GCA (large-vessel GCA) exist on an overlapping spectrum often sharing clinical phenotypes and outcomes, possibly reflecting different stages of disease.^121^ All three exhibit pleiotropic disease courses (monophasic vs relapsing and refractory) and could share mechanistic pathways relevant to disease persistence. For instance, studies have shown that macrophages and T-cells are important players in cranial-GCA and LV-GCA as well as PMR, yet research to date shows that B-cells appear to predilect the aorta.^122 123^ Similarly, IL-6 signatures have been linked to all three, however more clinical relapses are characteristically seen in LV-GCA patients.^32^ In addition, the disconnect between systemic and tissue inflammation further complicates the paradigm. Although this is an exciting perspective on potential biological disease endotypes, future studies should incorporate longitudinal designs with matched samples across time points of disease, but also access both tissue and peripheral blood to unravel the biology unifying or indeed distinguishing these clinical syndromes.

## Implications for therapy and future prospects

GCA therapeutics consist primarily of significant glucocorticoid exposure complicated by deleterious effects including infections, cardiovascular events, diabetes and osteoporosis.^124 125^ Although these are effective in controlling inflammation acutely, 34%–75% of patients do not achieve sustained remission in association with swift steroid tapering regimes.^8^ In parallel, studies employing repeat TA biopsies demonstrate active T-cell reactivity in >50% of cases, despite treatment.^85 87^ Multiple small studies investigating the role of IL-6 in GCA led to the repurposing of tocilizumab following clinical benefit via a phase 3 trial.^9 126^ This is currently the only biologic agent recommended by the British and European Rheumatology guidelines. Yet, sustained remission is only achieved in 42% of patients, while over one-third relapse following discontinuation, being exposed to additional glucocorticoid toxicity.^126^ Among the first randomised control trials in GCA, the evaluation of anti-TNFα therapy yielded negative results.^90 91^ Further, studies assessing the efficacy of IL-12/IL-23p40 blockade (ustekinumab) have been conflicting,^92 93^ despite its benefit in refractory GCA cases in the study by Conway *et al*.^92^ Other phase 3 trials are ongoing following successful phase 2 trials with GM-CSF receptor blockade (mavrilimumab), IL-17A blockade (secukinumab) and recombinant IgG CTLA-4 (abatacept), which appear promising in routine GCA management.^74 127 128^ Additional pathway inhibition has been explored including the JAK/STAT pathway, providing mixed outcomes.^129^ These relative treatment deficits reflect a knowledge gap, possibly related to what is increasingly understood in rheumatoid arthritis, where the immune compartment is no longer considered the sole driver of chronic inflammation and where stromal-immune interactions appear to be crucial in disease persistence. Fibroblast-targeting therapies are being explored in preclinical and early clinical trials with encouraging results.^130^ Modern investigative single-cell technologies will uncover key cellular and molecular players in GCA. Therapeutics in future could include directly targeting stromal cell derived effector molecules beyond IL-6, modulating stromal differentiation via inhibiting the driving signals (VEGF, IFN-γ), preventing their inflammatory reprogramming, or ultimately directly depleting the pathological subsets in refractory disease.
